# Coffee-based colloids for direct solar absorption

**DOI:** 10.1038/s41598-019-39032-5

**Published:** 2019-03-18

**Authors:** Matteo Alberghini, Matteo Morciano, Luca Bergamasco, Matteo Fasano, Luca Lavagna, Gabriele Humbert, Elisa Sani, Matteo Pavese, Eliodoro Chiavazzo, Pietro Asinari

**Affiliations:** 1 Department of Energy, Politecnico di Torino, Corso Duca degli Abruzzi 24, 10129 Torino, Italy; 2 Department of Applied Science and Technology, Politecnico di Torino, Corso Duca degli Abruzzi 24, 10129 Torino, Italy; 3 National Institute of Optics, National Research Council (CNR-INO), Largo E. Fermi 6, 50125 Firenze, Italy

## Abstract

Despite their promising thermo-physical properties for direct solar absorption, carbon-based nanocolloids present some drawbacks, among which the unpleasant property of being potentially cytotoxic and harmful to the environment. In this work, a sustainable, stable and inexpensive colloid based on coffee is synthesized and its photo-thermal properties investigated. The proposed colloid consists of distilled water, Arabica coffee, glycerol and copper sulphate, which provide enhanced properties along with biocompatibility. The photo-thermal performance of the proposed fluid for direct solar absorption is analysed for different dilutions and compared with that of a traditional flat-plate collector. Tailor-made collectors, opportunely designed and realized via 3D-printing technique, were used for the experimental tests. The results obtained in field conditions, in good agreement with two different proposed models, show similar performance of the volumetric absorption using the proposed coffee-based colloids as compared to the classical systems based on a highly-absorbing surface. These results may encourage further investigations on simple, biocompatible and inexpensive colloids for direct solar absorption.

## Introduction

Along the renewable backbone of the global energy mix, solar energy stands as one of the most promising resources to help reducing fossil fuel consumption and mitigate greenhouse gas emissions^1^. Current devices for solar energy conversion into thermal energy mostly rely on indirect absorption of the sunlight. This technology exploits a (selective) surface absorber with both high solar absorptance and low thermal emittance (i.e. low radiative heat loss), which efficiently captures the incident sunlight and then transfers the resulting thermal energy to a carrier fluid by conduction^2^. The efficiency of such indirect solar energy absorption is generally limited by the high surface temperature of the absorber with respect to that of the carrier fluid, which results in major convective heat losses with the surrounding environment. To overcome this issue, a promising alternative is represented by the direct absorption of sunlight, where a fluid serves both as solar energy absorber and heat carrier^3^. The advantage of this latter technique lies in the reduction of both convective and radiative heat losses, since the temperature peak is shifted from the absorber surface (indirect absorption) down to the bulk region of the carrier fluid (direct absorption)^4^. In this view, the solar absorbing fluid must have proper thermal and optical properties.

In a pioneering work, a *black fluid* consisting of 3.0 g/l of India ink in water was experimentally investigated for direct solar thermal energy absorption^5^. The observed performance was encouraging, and the potential of direct solar absorption in terms of technological simplification paved the way, in the later decades, to an intense research activity on such fluids. In recent years, the so-called *nanocolloids* (also known as *nanofluids*^6,7^), namely stable suspensions of nanoparticles in a base fluid, have attracted great attention for their use in direct solar absorption. These fluids are characterised by a suspended phase with the ability to confer specifically-enhanced photo-thermal properties to the base fluid^8–10^; thus, if opportunely designed, nanocolloids are expected to have a promising potential in solar-to-thermal energy conversion^11–13^.

A large number of different nanocolloids for solar energy conversion have been proposed^3,14^ and their properties extensively investigated^15–19^. Disagreement between experimental results in the literature has been reported and then investigated by several authors^20–22^, suggesting that more research is needed to clarify the underlying mechanisms at the basis of their modified properties. A considerable number of the studied nanocolloids rely on carbon nanoparticles dispersed in water or other liquids. Different nanoparticle types have been investigated, such as single- and multi-walled nanotubes^23–25^, graphite^26^, nano-horns^27–29^, or carbon powder in water^30^. However, the increasing use of carbon nanoparticles may lead to major environmental concerns^31^ and biological risks, because of their (cyto)toxicity^32,33^. In this sense, biocompatible (nano)colloids may represent a more sustainable and safe-by-design alternative to carbon-based nanosuspensions.

In this work, we propose an inexpensive, easy-to-prepare and environmentally-friendly colloid based on distilled water and Arabica coffee. This biocompatible colloid includes a small concentration of copper sulphate, to prevent biofouling, and glycerol, to lower the freezing point and allow its utilisation in cold climates. Optical characterization has been carried out for different dilutions, showing a good energy storage capability of the proposed fluid. Tailored collectors have been designed and realized, to test the photo-thermal performance of the coffee-based colloids as compared to classical indirect absorption based on a selective surface. Field tests, in good agreement with two different proposed models, show that the optimal dilution in terms of optical efficiency can be found as the one that guarantees the best energy storage capability during operation. Similar performance between the direct absorption using the proposed colloids and indirect absorption using a selective surface has been found, and this represents an encouraging result to a more in-depth engineering of the solar collectors for further enhancement of the conversion efficiency.

## Results and Discussion

This section illustrates, at first, the proposed coffee-based colloids and their optical characterization. Successively, we report on their photo-thermal performance (in terms of optical efficiency) obtained via experimental field tests and numerical models.

### Coffee-based colloids

**Figure 1:**
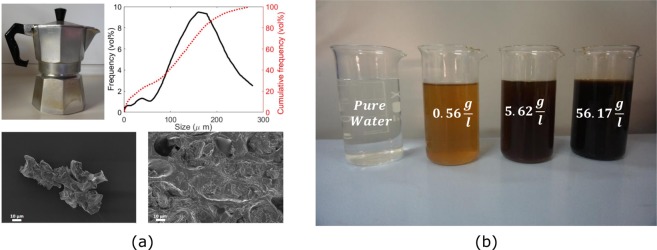
Synthesis of the coffee-based colloids. **(a)** Coffee pot *moka* used for the coffee preparation (top-left); size distributions of the suspended coffee particles (top-right); Scanning Electron Microscopy (SEM) images of the coffee particles (bottom). **(b)** Colloids with different G30 concentration (from right to left): pure G30 fluid (56.17 g/l of suspended particles); G30w10 fluid (10% dilution); G30w1 fluid (1% dilution in water); pure water.

Coffee is a complex substance, which shows various compositions and physical properties; in this work, a 100% Arabica Coffee is employed^34^. Among the different types of coffee makers, here we use *moka*, an aluminium coffee maker for stovetops available in different sizes (see Fig. 1(a), top-left). The adopted *moka* is made of a lower tank with 100 cm^3^ maximum capacity, a 35 cm^3^ capacity filter and a topper pot. The bottom tank is filled with water, while the filter tank with coffee. When the *moka* is heated up by a stove, the water contained in the lower tank starts to boil: the resulting pressure increase in the bottom tank drives the water to pass throughout the coffee in the filter, progressively filling up the upper pot^35^.

For the coffee preparation, 92 g of ultrapure water are introduced in the bottom tank, while 7.25 g of coarsely-ground coffee powder are put in the filter. The produced coffee is poured again in the lower tank, and the coffee prepared again. This protocol, known as the preparation of *student’s coffee*, allows to increase the amount of suspended particles and thus of caffeine dissolved in water. The resulting coffee (see the particle size distribution and Scanning Electron Microscopy (SEM) images in Fig. 1(a)) is then mixed with 30% wt. of pure glycerol, to lower the freezing temperature and allow outdoor utilisation also in cold climates. Clearly, the introduced glycerol affects also other thermophysical properties of the fluid^4,36^. Glycerol is chosen in place of ethylene glycol because of its non-toxicity. Finally, 2 ppm (parts per million) of copper sulphate (CuSO_4_) are added to reduce the risk of algae or moulds formation in the fluid. Five variants of the proposed colloid are considered: (i) the previously obtained solution, consisting of coffee, 30% wt. glycerol and 2 ppm CuSO_4_, which will be abbreviated as G30 from here onwards; (ii) a 1%, 10%, 20% and 50% volume fraction of G30 in distilled water, named respectively G30w1, G30w10, G30w20 and G30w50 (see Fig. 1(b) for the G30, G30w1 and G30w10 preparations). All the prepared coffee-based colloids demonstrated to be stable for the whole period of the experiments, that is, six months.

### Optical properties

**Figure 2:**
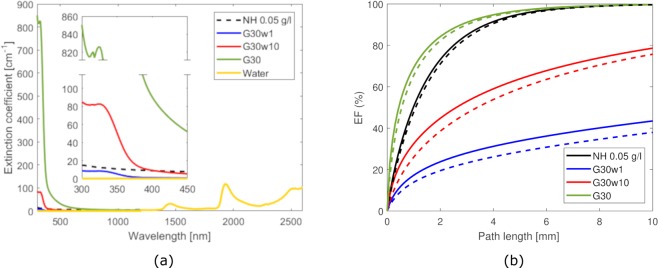
Optical properties of the coffee-based colloids (1%, 10% and 100% dilutions in water). (**a)** Comparison of the spectral extinction coefficient of the coffee-based colloids at different dilutions and a 0.05 g/l suspension of carbon nanohorns in water^27^. The G30 preparation (100% dilution) is coffee with 2 ppm of copper sulphate and 30% wt. glycerol; G30w1, G30w10 are respectively 1% and 10% volume fractions of G30 in distilled water. **(b)** Stored energy fraction (EF) as a function of the path length for the three considered coffee-based colloids. Solid lines correspond to the energy fraction obtained with Planck’s black body distribution, while dashed lines that obtained with the AM1.5 standard spectrum. The curves for a 0.05 g/l suspension of carbon nanohorns in water^27^ are also reported for comparison.

The optical properties of the proposed colloids are experimentally characterised in terms of extinction coefficient and stored energy fraction. The extinction coefficient is a characteristic optical property of the material, which is inversely related to the mean penetration distance of light into the medium. The extinction coefficient (*μ*_*λ*_) is defined as

1 $${\mu }_{\lambda }={\alpha }_{\lambda }+{\kappa }_{\lambda },$$

namely as the summation of absorption (*α*_*λ*_) and scattering (*κ*_*λ*_) coefficients at a given wavelength *λ*^37^. Fig. 2(a) shows the extinction coefficients for the G30, G30w1 and G30w10 colloids in the typical spectrum of the solar radiation (*λ* = 300–2600 nm). The results show an extremely intense optical extinction coefficient for the G30 fluid, reaching nearly 850 cm^−1^ at *λ* = 300 nm, and a slightly lower secondary peak at 324 nm. These features can be attributed to coffee, as pure water shows a high transparency in this spectral region. The height of these peaks decrease for increasing dilutions of G30 in water, as expectable. All fluids show a monotonically decreasing long tail with larger wavelengths, until all the curves overlap at around *λ* = 1200 nm, and present further peaks of extinction coefficient at *λ* = 1450, 1900 and beyond 2500 nm. The curve for a 0.05 g/l suspension of nanohorns in water^27^ is also reported for comparison.

The stored energy (or power) fraction can be computed as^27^

2 $$EF(x)=1-\frac{{\int }_{{\lambda }_{\min }}^{{\lambda }_{\max }}\,{I}_{\lambda }{e}^{-{\mu }_{\lambda }x}\,d\lambda }{{\int }_{{\lambda }_{\min }}^{{\lambda }_{\max }}\,{I}_{\lambda }d\lambda },$$

being *I*_*λ*_ the incident solar irradiance and *x* the penetration distance into the fluid (path length). Figure 2(b) shows the stored energy fraction calculated for the samples as a function of the path length. The minimum and maximum considered wavelengths of the solar spectrum are *λ*_*min*_ = 300 nm and *λ*_*max*_ = 2600 nm, respectively; whereas, the solar irradiance *I*_*λ*_ has been computed using both Planck’s black body distribution^38^ (considering the temperature of the sun to be 5,800 K and occupying a solid angle of 6.8 × 10^−5^ steradians) and, to provide the comparison, the AM1.5 standard spectrum with an overall power of 1000 W/m^2^. Note that the overall power in the considered range of wavelengths is recovered by an attenuation constant equal to either 0.77 (Planck’s black body) or 1.12 (AM1.5 standard spectrum). At *x* = 3 mm, for example, EF reaches 90% for G30 colloid, while it is 48% and 22% for G30w10 and G30w1 samples, respectively (in case of Planck’s black body distribution). The 0.05 g/l suspension of nanohorns in water^27^ presents an intermediate energy storage capability, being between the G30 and G30w10 colloids. For clarity, the same results for the G30w20 and G30w50 colloids are reported in a separated figure (Supplementary Fig. S1), where the G30w10 is also reported as a reference for comparison.

### Photo-thermal performance

**Figure 3:**
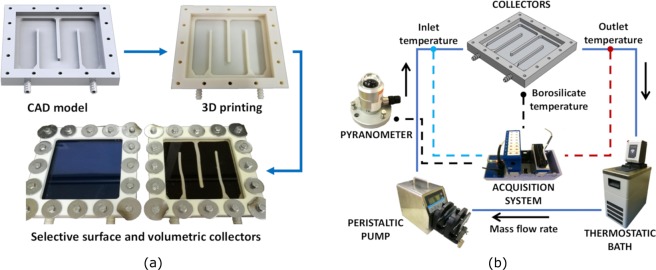
Set-up for the solar absorption tests. **(a)** Flow chart of the solar collectors design and manufacturing: from CAD model, to 3D-printed collector, to final assembly. During field tests, the performance of the direct solar absorber is compared with that of the traditional flat-plate collector. **(b)** Scheme of the experimental set-up used for testing the efficiency of the coffee-based colloids for the direct solar thermal energy absorption. Solid lines represent hydraulic pipes for the colloidal flow; dashed lines electric wires for data acquisition.

The photo-thermal performance of the coffee-based colloids is experimentally investigated and compared with that of a selective surface absorber using specifically-designed solar collectors (see Fig. 3(a)). The same geometry applies to study both direct and indirect absorption. In the former case, the colloid flows in the channel and directly absorbs the sunlight. In the latter case, a selective surface absorber is mounted on the collector and water flows through the underlying channels. A peristaltic pump is employed to provide a constant fluid flow through the collectors, and a thermostatic bath is used to control the inlet temperature of the fluids at the collectors (see Fig. 3(b)). The temperature jump between the inlet and outlet sections of the collectors (Δ*T*), the global solar irradiance (*I*_0_) and the ambient temperature (*T*_*a*_) are constantly monitored by an electronic acquisition system. The optical efficiency of the collectors is then computed as

3 $${\eta }_{o}=\frac{\dot{m}\,{c}_{p}\,{\rm{\Delta }}T+{\dot{Q}}_{loss}}{{A}_{net}\,{I}_{0}},$$

being $\dot{m}=\rho \dot{V}$ the mass-flow rate, *c*_*p*_ the specific heat of the considered fluids (coffee-based colloids or water, for direct and indirect absorption respectively), ${\dot{Q}}_{loss}$ the global thermal losses and *A*_*net*_ the net exposed surface. Note that this latter area is approximately 10% lower in case of direct absorption, as it corresponds to the net exposed surface of the flowing fluids with respect to that of the exposed selective surface (compare Fig. 3(a), bottom). Thermal losses are obtained through an equivalent thermal network model based on electrical analogy (see Section *One-dimensional model* in *Methods*), while the optical efficiency through energy conservation for the two cases (see Supplementary Fig. S2). Experimental tests are carried out in the same conditions for direct and indirect absorption, and the efficiency of the collectors compared.

**Figure 4:**
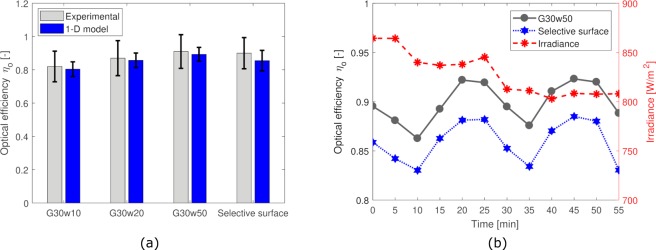
Photo-thermal performance. (**a)** Results obtained for the optical efficiency of the proposed coffee-based colloids at different dilutions (10%, 20% and 50% G30 volume fraction in water) and of the selective surface absorber. The average value obtained at steady state (5 minutes sampling frequency) for three different flow rates (0.138, 0.276 and 0.414 ml/s) is reported. The error bars have been obtained via uncertainty quantification on the experimental data and on the model parameters. **(b)** Time evolution of the experimental optical efficiency of the G30w50 fluid (black), of the selective surface (blue) and of the irradiance (red) for the experimental test at 0.138 ml/s flow rate. The experimental tests have been carried out at Politecnico di Torino (Turin, Italy) on 20 June 2018.

The colloids are tested at three different flow rates, namely 0.138, 0.276 and 0.414 ml/s, using the experimental set-up shown in Supplementary Fig. S3. The fluid reservoirs are kept at constant temperature (25 °C) using the thermostatic bath. The time evolution of the systems (temperature at the inlet and outlet sections of the collectors as well as the ambient conditions) is constantly monitored. Since the tested temperatures of the fluids never exceeded 40 °C, vapor formation was never observed in the experiments, demonstrating no influence of this physical phenomenon on the optical efficiency. Steady-state data are considered for the post-processing (averaging every 5 minutes). The results obtained are shown in Fig. 4(a), where the mean optical efficiency over the three analyzed flow rates is reported for the G30w10, G30w20 and G30w50 fluids and for the selective surface absorber. The volumetric receiver performs similarly to the surface absorber, with the best case being the volumetric absorption using the G30w50 colloid. The numerical model, which takes the outlet temperature as a variable (instead of the measured value), shows very good agreement with the experimental results, which are within the error bar for all the analyzed cases (see Section *Uncertainty quantification* in *Methods* for details). The mean and maximum relative errors obtained by the model on the outlet temperature are respectively 2% and 4% with respect to the experimental values. Figure 4(b) shows the instantaneous efficiency obtained during 55 minutes of the field test for the G30w50 fluid at 0.138 ml/s mass-flow rate.

### Numerical predictions

Numerical models have been developed and validated against the experimental data. In particular, we developed: (i) a one-dimensional model based on electrical analogy; and (ii) a two-dimensional Computational Fluid Dynamics (CFD) model. Outlet fluid temperatures are very well recovered in all cases, with an average 2% and maximum 6% relative errors with respect to the experimental data. Globally, the major source of error (7% maximum) is found on the temperature of the borosilicate glass, which affects global losses towards the ambient and thus the optical efficiency of the collectors.

Having validated the two models, we perform a numerical study for constant ambient temperature (*T*_*in*_ = *T*_*a*_ = 25 °C), irradiance (*I*_0_ = 1000 W/m^2^) and wind speed (*v*_*w*_ = 1 m/s), to assess the performance of the collectors independently from variable external conditions. The results obtained, coherently with the experimental evidences, show increasing power gain in all cases with the liquid flow rate, as well as with the G30 volume fraction for the volumetric absorption (see Supplementary Fig. S4).

**Figure 5:**
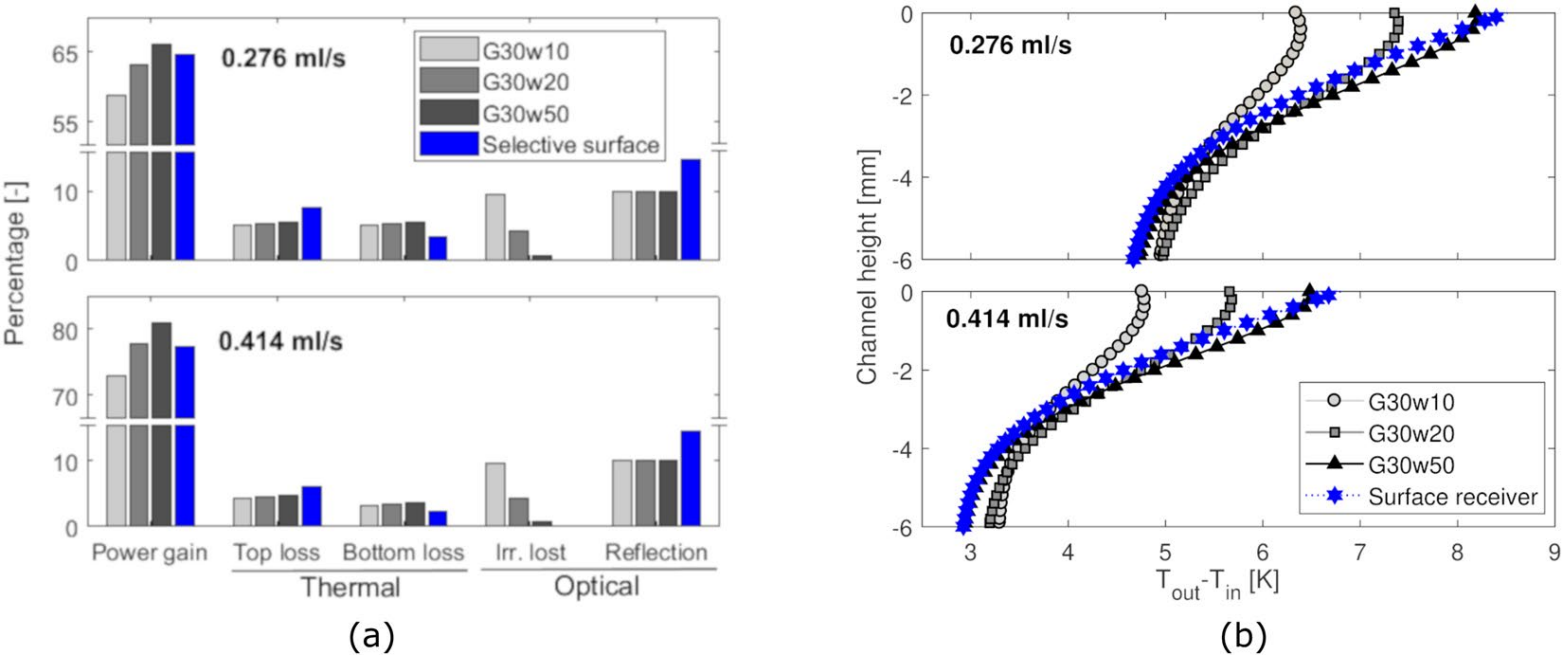
Modeling of thermal performances. **(a)** Decomposition and analysis of the power components (1D model) for the different configurations (direct and selective surface absorption) at 0.276 ml/s (top histogram) and 0.414 ml/s (bottom histogram) flow rates. Higher fluid speed reduce the thermal losses towards the environment due to lower operating temperatures. The irradiance absorption is not influenced by different mass flow rates, hence the design favours the fluid able to capture as high irradiance as possible, namely the G30w50 fluid. **(b)** Fluid temperature profiles at the outlet section (inlet temperature is constant) obtained with the 2D model. The colloids have lower surface temperature than that of the surface receiver, and top thermal losses are lower. Lower fluid concentrations lead to reduced surface temperature and less sharp profiles.

An analysis of the various loss terms for the volumetric and surface absorbers shows that convective thermal losses are more important at low liquid-flow rates, due to the higher average fluid temperature (see Fig. 5(a,b)). On the other hand, optical losses does not depend on the flow rate, but on the optical properties of the flowing fluids and of the materials of which the collector is made. Part of the incoming radiation is reflected by the borosilicate glass and by the selective surface in case of indirect absorption; another part is either directly absorbed by the fluid (according to its extinction coefficient) or absorbed/reflected again by the material at the bottom of the channel. If absorbed, this latter radiation heats up the bottom layer, and thus, the fluid by convection; if reflected, it is partly absorbed by the fluid in a second pass and partly lost to the ambient. This balance between absorption or reflection at the bottom of the channel plays an important role on the efficiency of the collector; thus, thermal performance can be optimized by opportunely tuning the geometry of the channel with respect to the optical properties of the flowing fluid and of the material the collector is made of.

## Conclusions

The proposed coffee-based colloids have shown competitive optical and thermal properties for direct solar absorption. Field tests, in good agreement with numerical models, have demonstrated that these fluids can provide similar performance to the traditional indirect absorption based on selective surfaces. In particular, the optimal dilution in terms of optical efficiency of the coffee-based colloids has been found as the one that guarantees the best energy storage capability during operation. One- and two-dimensional models have been validated against the experimental data, within an average 2% and maximum 6% relative errors. These models have been used to analyse different operating conditions, and can be readily employed to explore also different fluids and materials to reduce losses and optimize the efficiency. Moreover, an extension of the two-dimensional to three dimensions would also allow to optimize the collector design. These results may pave the way to a new, unconventional family of biocompatible, environmentally sustainable and inexpensive colloids for solar applications, for example suited for vapor formation^39,40^, seawater desalination^41,42^, domestic hot water production^43,44^, or sustainable solar cooling^45,46^.

## Methods

### Thermal and optical characterisations

The specific heat capacity of the G30 fluid has been measured at the Department of Applied Science and Technology (DISAT) of Politecnico of Torino, using Differential Scanning Calorimetry (DSC). This technique allows to obtain the specific heat capacity as a function of temperature for the tested sample^47^. Tests are performed at atmospheric pressure and for temperatures ranging from 10 to 80 °C. Linear fitting of the experimental results yields *c*_*p*_(*T*) = 9.6089 *T* +3501.8, with coefficient of determination *R*^2^ = 0.997. For water, previously reported values have been adopted for the specific heat as a function of temperature^48^. For the different dilutions of the G30 fluid in water, weighted means are considered.

The extinction coefficient of the proposed colloids has been computed at the National Institute of Optics (CNR-INO) in Florence. Coffee presents a very complex chemical composition, which includes e.g. polysaccharides, proteins, lipids and lignin^49^, with a slight sedimentation generally observed^50^. In order to avoid any effect of unstable (micro-nano)particles on the optical properties, fluids were left sedimenting for two days before the characterization. The spectral transmittance is firstly measured using a double-beam UV-VIS-NIR spectrophotometer *PerkinElmer Lambda900* with variable cell length. The spectral extinction coefficient is then calculated from the experimental transmittance via Beer-Lambert law using a method that allows to correctly remove unwanted reflectance artefacts^51,52^.

### Collector design and manufacturing

The solar thermal collectors used for field experiments have been manufactured at the Department of Energy (DENERG) of Politecnico of Torino. The collectors are designed using a CAD software, see Supplementary Fig. S5. The collector has an overall exposed surface equal to 100 cm^2^; whereas, the channels for the fluid flow are 6 mm deep and 22.5 mm wide, and the effective exposed surface is 90.6 cm^2^. The same collector geometry is employed for both direct solar absorbtion and indirect surface absorption. In the former case, the channels are filled with the coffee-based colloid and are covered by a 6.5 mm thick borosilicate glass (*BOROFLOAT*^*®*^, Edmund Optics), while in the latter case they are filled with water (heat carrier fluid) and covered by a selective surface absorber (*TiNOX*^*®*^ *Energy*, Almeco) and the borosilicate glass on top. *TiNOX*^*®*^ *Energy* is an optimised material able to reduce radiative heat losses thanks to a low thermal emission coefficient (4%) in the infrared range, while providing 94% absorption in the range of solar wavelengths. The collectors are manufactured by means of 3D-printing technique, using ABS filaments (*Elite*, Stratasys). In order to reduce the porosity of the ABS plastic and avoid fluid leakages, *Nano-Seal 180 W* is applied to the prototypes. A tailored silicon gasket (*GLS-50* and 5% *T-30* catalyst, Prochima) is molded and, after a one-day stand at ambient temperature, assembled between the fluid channels and the borosilicate glass of the volumetric absorber. A flexible framework produced in TPU 95 A via 3D-printing (*Ultimaker 3*) is positioned on top of the borosilicate glass, to avoid the risk of cracking during the closure of the assembly, which is realized using 16 M5 bolts (compare Fig. 3(a)).

### Field tests

The different dilutions of the colloids are prepared in terms of the G30 volume fraction in water *ϕ* = *V*_*G*30_/(*V*_*G*30_ + *V*_*w*_), from which, the required mass of the G30 fluid is easily obtained as

4 $${m}_{G30}=\frac{\varphi }{1-\varphi }\frac{{\rho }_{G30}}{{\rho }_{w}}\,{m}_{w}\mathrm{.}$$

The experimental density of the G30 fluid is *ρ*_*G*30_ = 994 kg/m^3^, assumed constant with respect to temperature. The density of water (*ρ*_*w*_) has been computed using the equation reported by Kell^48^ at room temperature (*T*_*a*_ = 20 °C). The density of the dilutions is computed as a weighted mean of the components. Temperatures are sampled using K-type thermocouples, connected to a data acquisition system (*NI-9212*, National Instruments). In the experimental set-up, the ambient temperature and that of the glass cover are constantly monitored, besides the inlet and outlet temperatures of the flowing fluids. The wind speed is provided by the meteorological station at Politecnico di Torino. The fluids are pumped through the collectors by a peristaltic pump (*BT100S*, Lead Fluid), which provides a constant volumetric flow rate within a 0.5% error. A thermostatic bath (*CORIO CD-300F*, Julabo) is used to control the inlet temperature of the fluids at the collectors. Thermo-physical properties of the fluids are evaluated at the average temperature between inlet and outlet sections of the collectors.

### One-dimensional model

The solar collectors can be modeled as an equivalent one-dimensional thermal network. Based on electrical analogy, each loss term is modeled as a thermal resistance. Starting from the top layer of the prototype, the convective and radiative losses from the borosilicate glass cover to the ambient are modeled as parallel resistances. The convective heat transfer coefficient between ambient and glass cover is evaluated as^53^

5 $${h}_{c,a}^{c}=0.86\,{{\rm{Re}}}^{\mathrm{1/2}}\,{{\rm{\Pr }}}^{\mathrm{1/3}}\,\frac{{k}_{a}({T}_{a})}{{D}_{h}},$$

where Re and Pr are the Reynolds and Prandtl numbers of air, *D*_*h*_ the hydraulic diameter of the glass cover and *k*_*a*_ the thermal conductivity of air. The radiative heat-transfer coefficient between the glass cover and the sky is evaluated as

6 $${h}_{c,a}^{r}={\varepsilon }_{c}\sigma ({T}_{c,u}^{2}+{T}_{sky}^{2})({T}_{c,u}+{T}_{sky}),$$

being *ε*_*c*_ the infra-red emission coefficient of the cover (black body approximation), *T*_*c*,*u*_ its average upper temperature, *T*_*sky*_ = −25 °C and *σ* the Stefan-Boltzmann constant. The conductive resistance in the glass cover is *s*_*c*_/*k*_*c*_ (being *s*_*c*_ and *k*_*c*_ the thickness and the thermal conductivity of the borosilicate glass cover, respectively). Assuming the lateral walls of the channel as adiabatic and a constant heat flux condition at the upper and lower boundaries, the Nusselt number for the laminar flow of the fluid through the rectangular channel is Nu = 8^54^. The convective heat-transfer coefficient between the solid walls and the fluid can be then computed as

7 $${h}_{f,w}^{c}={\rm{Nu}}\,\frac{{k}_{f}({T}_{f})}{{D}_{h}},$$

where *T*_*f*_ is the average fluid temperature, *k*_*f*_(*T*_*f*_) the thermal conductivity of the fluid and *D*_*h*_ the hydraulic diameter of the channel. All thermodynamic properties are averaged along the length of the channel.

#### Volumetric absorption

For the volumetric absorber, the equivalent thermal transmittance at the top side of the receiver is then computed as

8 $${U}_{top}^{vol}={(\frac{1}{{h}_{c,a}^{c}+{h}_{c,a}^{r}}+\frac{{s}_{c}}{{k}_{c}}+\frac{1}{{h}_{f,w}^{c}})}^{-1}\mathrm{.}$$

The radiative source term *S*_*irr*_ is obtained from a simplified version of the Radiative Transfer Equation (RTE), which neglects the contribution of scattering and thermal emission^38^, as

9 $${S}_{irr}={\tau }_{c}[{\int }_{{\lambda }_{\min }}^{{\lambda }_{\max }}\,{I}_{\lambda }(1-{e}^{-{\mu }_{\lambda }H})\,d\lambda +(1-{\alpha }_{abs}){\int }_{{\lambda }_{\min }}^{{\lambda }_{\max }}\,{I}_{\lambda }{e}^{-{\mu }_{\lambda }H}(1-{e}^{-{\mu }_{\lambda }H})d\lambda ],$$

where *λ*_*min*_ = 300 nm and *λ*_*max*_ = 2600 nm are the minimum and maximum wavelengths analysed, *τ*_*c*_ is the transmission coefficient of the glass cover, *I*_*λ*_ the irradiance corresponding to the Plank distribution for a black body^55^ and *H* the height of the channel. The first term on the right-hand side accounts for the absorption during the first pass, while the second for the absorbed radiation which results from the reflection at the bottom of the channel (1 − *α*_*abs*_ with *α*_*abs*_ = 0.4). Finally, the model equation is

10 $$\dot{m}\,{c}_{p}\frac{d{T}_{f}}{dx}=-\,{U}_{top}^{vol}\,W\,({T}_{f}-{T}_{a})-{h}_{f,w}^{c}\,W({T}_{f}-{T}_{w,l})+W\,{S}_{irr},$$

being *W* the width of the channel and *T*_*w*,*l*_ the average temperature of the ABS material at the bottom. Since *T*_*w*,*l*_ is initially unknown, Eq. 10 is solved iteratively while imposing the following energy conservation at the bottom side of receiver:

11 $${\alpha }_{abs}\,{\tau }_{c}{\int }_{{\lambda }_{\min }}^{{\lambda }_{\max }}\,{I}_{\lambda }{e}^{-{\mu }_{\lambda }H}\,d\lambda +{h}_{f,w}^{c}\,({T}_{f}-{T}_{w,l})-{U}_{bot}^{vol}({T}_{w,l}-{T}_{a})=0,$$

where

12 $${U}_{bot}^{vol}={(\frac{{s}_{abs}}{{k}_{abs}}+\frac{1}{{h}_{c,a}^{c}})}^{-1}$$

is the equivalent thermal transmittance from the ABS material to the bottom ambient. In Eq. 12, the conductive resistance of the ABS material *s*_*abs*_/*k*_*abs*_ (being *s*_*abs*_ and *k*_*abs*_ the thickness and the thermal conductivity of the ABS material, respectively) and the convection from the ABS to the ambient ${h}_{c,a}^{c}$ are considered.

#### Surface absorption

For the surface absorber, the surface-to-cover convective and radiative coefficients are treated as parallel resistances. The convective effect ${h}_{s,c}^{c}$ is recovered from a previously reported correlation for natural convection^53^ and the radiative effect is obtained considering two facing surfaces as

13 $${h}_{s,c}^{r}=\frac{\sigma ({T}_{c,l}^{2}+{T}_{s}^{2})({T}_{c,l}+{T}_{s})}{\mathrm{1/}{\varepsilon }_{s}+\mathrm{1/}{\varepsilon }_{c}-1},$$

where *T*_*c*,*l*_ and *T*_*s*_ are respectively the lower temperature of the cover and of the surface, *ε*_*s*_ is the infra-red emission coefficient of the surface. Again, the conductive resistance of the ABS material and the convection from the ABS to the ambient are considered. Thus, the equivalent transmittances towards the ambient are

14 $${U}_{top}^{surf}={(\frac{1}{{h}_{c,a}^{c}+{h}_{c,a}^{r}}+\frac{{s}_{c}}{{k}_{c}}+\frac{1}{{h}_{s,c}^{c}+{h}_{s,c}^{r}})}^{-1},$$

15 $${U}_{bot}^{surf}={(\frac{1}{{h}_{f,w}^{c}}+\frac{{s}_{abs}}{{k}_{abs}}+\frac{1}{{h}_{c,a}^{c}})}^{-1}.$$

In this case, the source term is *S*_*irr*_ = *I*_0_*τ*_*c*_*α*_*s*_, being *α*_*s*_ = 0.95 the absorption coefficient of the selective surface in the solar spectrum. In order to model the surface-to-fluid heat transfer, we define an efficiency factor as^53^

16 $${F}^{{\rm{^{\prime} }}}={[{U}_{top}^{surf}D({({U}_{top}^{surf}W+{U}_{top}^{surf}F(D-W))}^{-1}+{(\pi {D}_{h}{h}_{f,w}^{c})}^{-1})]}^{-1},$$

where *D* = *A*/*L* (with *L* = 404 mm being the length of the channel, equal to the full fluid path through the collector) is the inter-axial distance between two adjacent channels and *F* is the standard fin efficiency (see reference^53^ for details). The model equation for this case yields

17 $$\dot{m}\,{c}_{p}\frac{d{T}_{f}}{dx}=-\,{F}^{{\rm{^{\prime} }}}{U}_{top}^{surf}D({T}_{f}-{T}_{a})-{U}_{bot}^{surf}W({T}_{f}-{T}_{a})+D\,{F}^{{\rm{^{\prime} }}}\,{S}_{irr}.$$

The two models are implemented and solved iteratively in MATLAB^®^ over the channel length. The mean and maximum relative errors obtained by the model on the outlet temperature are respectively 2% and 4% with respect to the experimental values.

### Two-Dimensional Model

A two-dimensional model of the collectors is set-up using COMSOL^®^ Multiphysics 5.3. The length of the fluid domain (*L*) is equal to the full fluid path through the collector (404 mm), and the height (*H*) equal to that of the channel (6 mm). The width (*W*), used as a reference for pre- and post-processing, is equal to that of the channel (22.5 mm). The steady, incompressible Navier-Stokes-Fourier system is solved, to obtain the temperature field.

#### Volumetric absorption

For the volumetric absorption (radiation in participating media), the energy equation reads as

18 $${\rho }_{f}\nabla \cdot ({c}_{p,f}(T-{T}_{0})\,\,{\bf{u}})=\nabla \cdot ({k}_{f}\nabla T)+{S}_{irr},$$

being *ρ*_*f*_ the density of the fluid, *c*_*p*,*f*_ and *k*_*f*_ respectively its specific heat and thermal conductivity, and *S*_*irr*_ a volumetric source term which accounts for the incoming irradiance. This latter term corresponds to the divergence of the radiative flux and, in general, is obtained by solving the Radiative Transfer Equation (RTE)^38^ in the domain; however, in this study the incident solar radiation is considered collimated (and scattering is neglected), and the source term is obtained as

19 $${S}_{irr}=-\,\nabla \cdot {{\bf{q}}}_{irr}=-\,\frac{d}{dy}{\int }_{{\lambda }_{\min }}^{{\lambda }_{\max }}\,{I}_{\lambda }[{e}^{-{\mu }_{\lambda }y}+\mathrm{(1}-{\alpha }_{abs}){e}^{-{\mu }_{\lambda }(2H-y)}]\,d\lambda ,$$

being *I*_*λ*_ the reference AM1.5 solar spectrum, *α*_*abs*_ the absorption coefficient of the fluid (considered as a gray medium^4^) and *μ*_*λ*_ the extinction coefficient. In this case, we adopt a mean value for the extinction coefficient obtained as

20 $$\bar{\mu }=\frac{{\int }_{{\lambda }_{\min }}^{{\lambda }_{\max }}\,{I}_{\lambda }{\mu }_{\lambda }d\lambda }{{\int }_{{\lambda }_{\min }}^{{\lambda }_{\max }}\,{I}_{\lambda }d\lambda },$$

with *λ*_*min*_ = 300 nm and *λ*_*max*_ = 2600 nm. The effect of the borosilicate glass cover on top of the domain and of the ABS material at the bottom of the channel are modeled using appropriate boundary conditions:

21 $${{k}_{a}\frac{\partial T}{\partial y}|}_{y=0}={(\frac{1}{{h}_{f,a}^{c}+{h}_{f,a}^{r}}+\frac{{s}_{c}}{{k}_{c}})}^{-1}({T}_{y=0}-{T}_{a});$$

22 $${{k}_{a}\frac{\partial T}{\partial y}|}_{y=H}={(\frac{1}{{h}_{f,a}^{c}}+\frac{{s}_{abs}}{{k}_{abs}})}^{-1}({T}_{y=H}-{T}_{a});$$

where ${h}_{f,a}^{c}$ and ${h}_{f,a}^{r}$ are respectively the convective and radiative heat transfer coefficients between the fluid and the ambient. These coefficient are computed similarly to those in Eqs (5) and (6); however, in this case, the fluid-to-cover radiation has been included into the fluid-to-ambient term (*ε*_*f*_ = 1) and that radiative losses are neglected from the ABS material to the ambient. The terms *s*_*c*_/*k*_*c*_ and *s*_*abs*_/*k*_*abs*_ account for the conductive resistances of the borosilicate glass cover and of the ABS material respectively.

#### Surface absorption

For the surface absorption, the solar radiation is partly reflected and partly absorbed by the selective surface. Heat is transferred to the fluid by conduction, so Eq. (18) does not include the source term. The boundary conditions of Eqs (21) and (22) still hold, except that the spectral emissivity of the selective surface is *ε*_*s*_ = 0.04 in the radiative loss term.

The domain is discretized using a proper number of quadratic cells (25,800) and a direct solver, namely the Parallel Direct Sparse Solver (PARDISO) is adopted to numerically solve the governing equations. Independence of the numerical solution from the computational grid has been verified.

The mean and maximum relative errors obtained by the model on the outlet temperature are respectively 2% and 6% with respect to the experimental values.

### Uncertainty quantification

The following two types of uncertainties are considered for the analysis of the experimental data^56^: statistical uncertainty of the experimental measurements (A-type), and uncertainty due to the offset/calibration of the experimental instruments (B-type). The population mean and standard deviation are computed from the statistical samples using the Student’s t-distribution and Chi-squared distribution^57^; thus, the A-type uncertainty on the optical efficiency of Eq. (3) is computed as

23 $${\sigma }_{{\eta }_{o},A}={\sigma }_{{\eta }_{o}}\sqrt{\frac{{t}_{1-\alpha \mathrm{/2}}^{2}}{n}+\frac{n-1}{{\chi }_{\alpha \mathrm{/2}}^{2}}},$$

being ${\sigma }_{{\eta }_{o}}$ the standard deviation of the sample and *n* the number of independent measurements (that is, *n*−1 degrees of freedom with significance level *α* = 0.05). For the B-type uncertainty, we consider the offset/calibration error on the input measurements for the following independent quantities *ψ* = {*T*_*in*_, *T*_*out*_, *T*_*a*_, *I*_*o*_, *v*_*w*_}. Then the B-type uncertainty is computed as^57^

24 $${\sigma }_{{\eta }_{o},B}=\sqrt{\sum _{i=1}^{5}{({{\rm{\Delta }}}_{{\psi }_{i}}\frac{\partial {\eta }_{o}}{\partial {\psi }_{i}})}^{2}},$$

being ${{\rm{\Delta }}}_{{\psi }_{i}}$ the standard uncertainty on the independent quantities *ψ*_*i*_. According to the supplier datasheets, the standard errors are: ±0.4 °C on temperatures (*T*_*in*_, *T*_*out*_, *T*_*a*_) due to the offset of the thermocouples; ±5% on the irradiance (*I*_0_) due to the offset of the pyranometer. In order to be conservative, we also assume quite a large uncertainty on the wind speed provided by the meteorological station at Politecnico di Torino, that is ±0.5 m/s. Finally, the global uncertainty (which is shown by the error bars on the experimental data in Fig. 4(a)) is obtained as^57^

25 $${\sigma }_{{\eta }_{o}}=\sqrt{{\sigma }_{{\eta }_{o},A}^{2}+{\sigma }_{{\eta }_{o},B}^{2}}\mathrm{.}$$

In the same Fig. 4(a), the error bars of the 1D model are reported. In this case, we adopt a standard uncertainty quantification (UQ) procedure. Uncertainty is assumed on the optical properties, that is, on the absorption coefficient of the ABS (*α*_*abs*_ ± 15%), of the selective surface (*α*_*s*_ ± 5%) and on the reflection coefficient of the borosilicate glass (*τ*_*c*_ ± 5%). The former uncertainty on the ABS material has been assumed in a conservative way based on typical values reported for white plastics^58^, while the latter two uncertainties have been adopted based on the supplier datasheets. The uncertainties are provided after feeding 1,500 normally-distributed random inputs to the 1D model, and the standard deviation of the model output distribution is shown by the error bars of the model in Fig. 4(a).

## Supplementary information

Supplementary information.

Stability of coffee-based colloids.
