# Supplementary material for: Coffee-based colloids for direct solar absorption
Source: Sci Rep. 2019 Mar 18;9:4701. doi: 10.1038/s41598-019-39032-5 (PMC6423041; doi:10.1038/s41598-019-39032-5)
Supplement: Supplementary file 1 — Supplementary information. [file 41598_2019_39032_MOESM1_ESM.pdf]

Coffee-based colloids for direct solar absorption

Matteo Alberghini[1], Matteo Morciano[1], Luca Bergamasco[1], Matteo Fasano[1], Luca Lavagna[2], Gabriele Humbert[1], Elisa Sani[3], Matteo Pavese[2], Eliodoro Chiavazzo[1], and Pietro Asinari[1,*]

[1]Department of Energy, Politecnico di Torino, Corso Duca degli Abruzzi 24, 10129 Torino, Italy
[2]Department of Applied Science and Technology, Politecnico di Torino, Corso Duca degli Abruzzi 24, 10129 Torino, Italy
[3]National Institute of Optics, National Research Council (CNR-INO), Largo E. Fermi 6, 50125 Firenze, Italy
[*]Corresponding author: pietro.asinari@polito.it

Supplementary information

Supplementary Figures

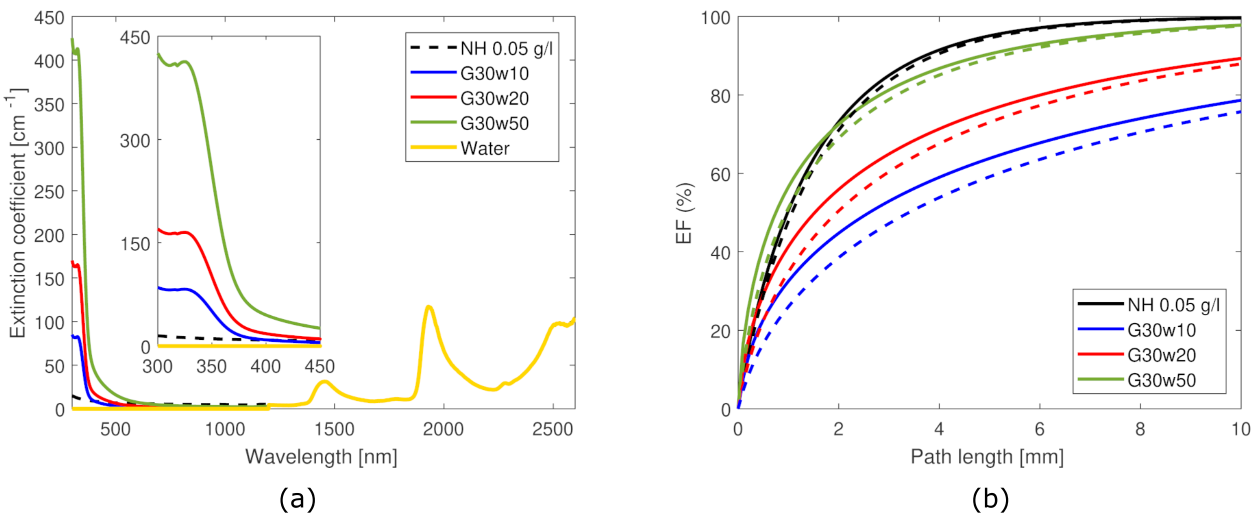

Figure S1. Optical properties of the coffee-based colloids (10%, 20% and 50% dilutions in water). (a) Comparison of the spectral extinction coefficient of the coffee-based colloids at different dilutions (G30w10, G30w20, G30w50) and a 0.05 g/l suspension of carbon nanohorns in water.[1] **(b)** Stored energy fraction (EF) as a function of the path length for the three considered coffee-based colloids. Solid lines correspond to the energy fraction obtained with Planck's black body distribution, while dashed lines that obtained with the AM1.5 standard spectrum. The curves for a 0.05 g/l suspension of carbon nanohorns in water[1] is also reported for comparison.

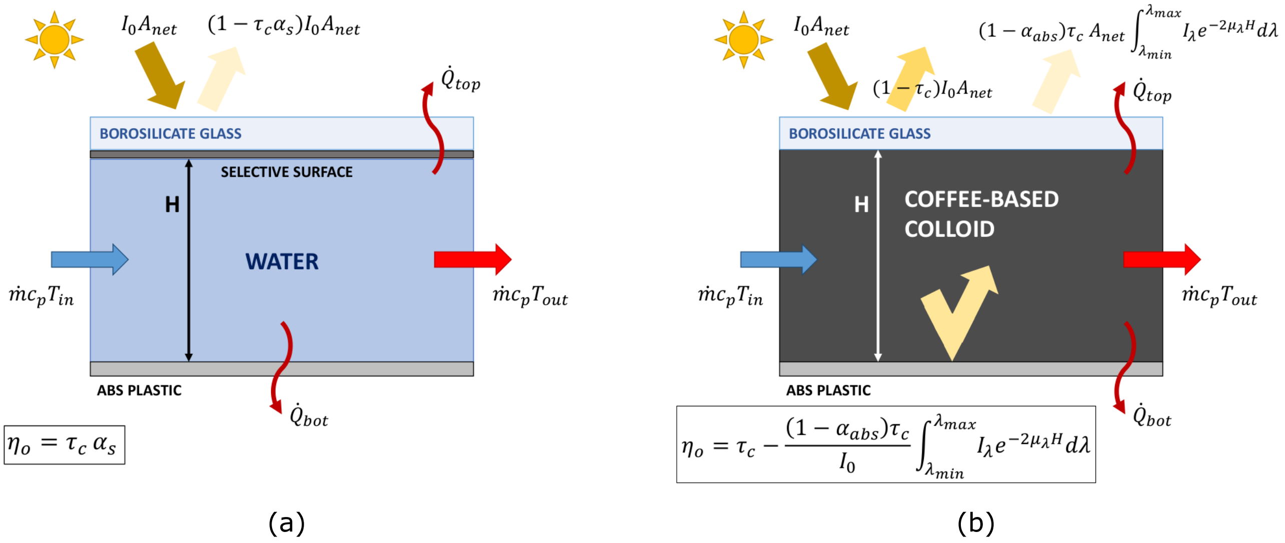

Figure S2. **Energy conservation in the solar collectors.** **(a)** Energy conservation for the selective surface absorption. **(b)** Energy conservation for the direct absorption using the proposed colloids. The expression of the theoretical optical efficiency (η_o) is reported for the two cases. See Section *Methods* for a detailed description of the symbols.

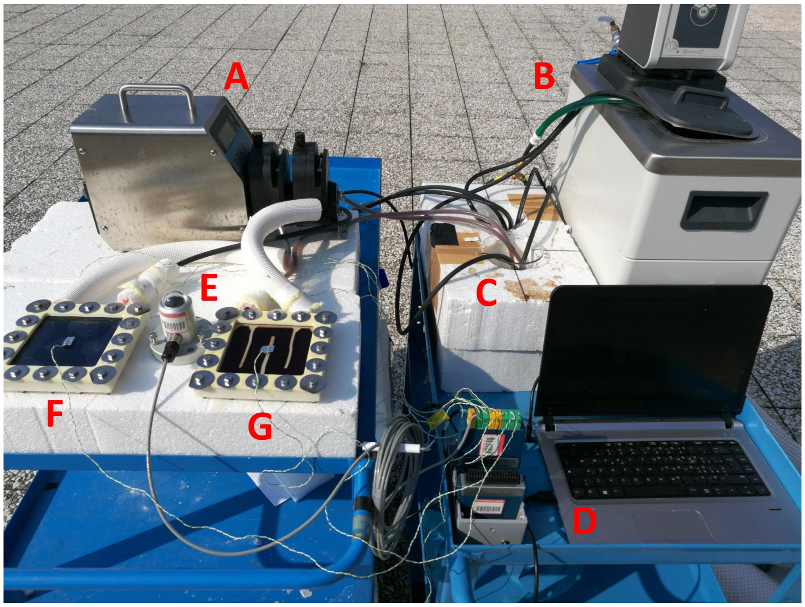

Figure S3. Experimental set-up for field tests. The experimental system consists of: **A** peristaltic pump, **B** thermostatic bath, **C** insulated fluid reservoirs, **D** acquisition system, **E** pyranometer, **F** selective surface receiver, **G** volumetric receiver. During tests, all the connections and acquisition systems are protected from the disturbing effects of the sunlight and wind using a proper insulating cover.

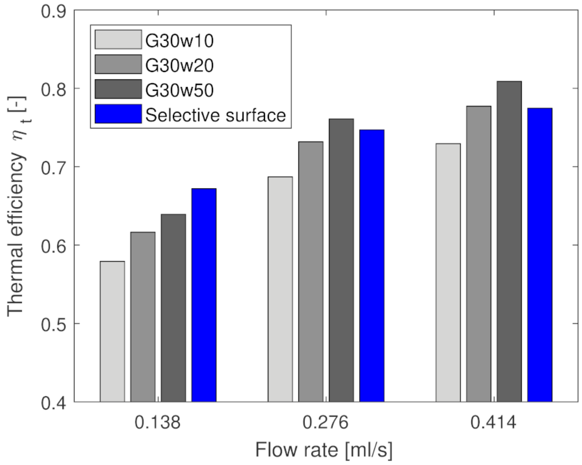

(a)

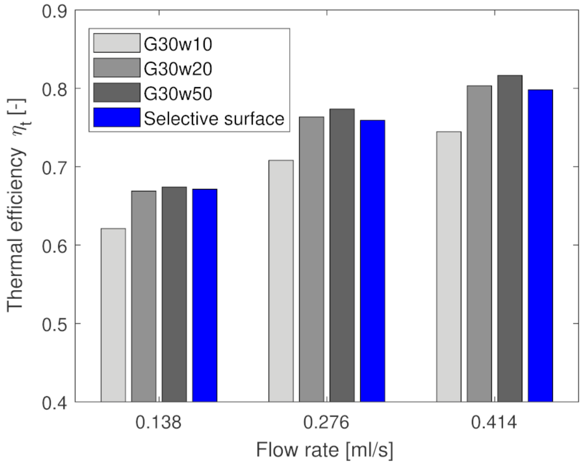

(b)

Figure S4. Comparison of the 1D and 2D model results. Comparison of the thermal efficiency (defined as $\eta_t = \dot{m}c_p\Delta T/(I_0 A_{net})$) obtained with the **(a)** 1-D model and **(b)** 2-D model in controlled conditions ($T_a = T_{in} = 25$°C and I_0 =1000 W/m^2 total irradiance). Different fluid dilutions at different flow rates are compared with the selective surface absorber. The results obtained with the two models are in good agreement.

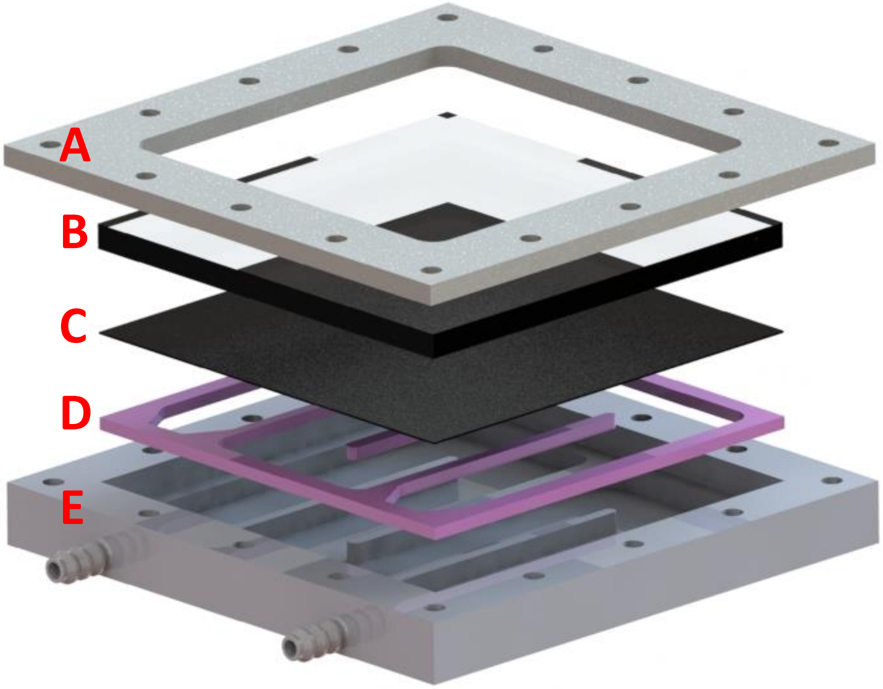

Figure S5. Rendering of the CAD model for the collectors. The CAD model of the traditional flat-plate solar thermal collector is represented with an exploded view. The following parts are shown (from top to bottom): **A** plastic frame, **B** borosilicate glass, **C** selective surface absorber, **D** silicon gasket, **E** channels for the fluid flow. Note that the assembly for direct solar absorption with the proposed colloids does not include the selective surface absorber (**C**).

References

1. Sani, E. *et al.* Carbon nanohorns-based nanofluids as direct sunlight absorbers. *Opt. Express* **18**, 5179–5187 (2010).
